# The effectiveness of interventions for offending behaviours in adults with autism spectrum disorders (ASD): a systematic PRISMA review

**DOI:** 10.1186/s40359-024-01770-1

**Published:** 2024-05-30

**Authors:** Jody Salter, Sarah Blainey

**Affiliations:** 1 Department of Forensic and Neurodevelopmental Sciences, Institute of Psychiatry, Psychology and Neuroscience, King’s College London, London, UK; 2 School of Health and Society, University of Salford, Greater Manchester, UK; 3 Central and North West London NHS Foundation Trust, London, UK

**Keywords:** Autism spectrum disorder, Offending behaviour, Forensic Psychiatry, Forensic psychology, Intervention, Recidivism, Criminal justice system

## Abstract

Previous research has suggested that the core features of autism spectrum disorders (ASD) may contribute to offending behaviours and increased vulnerability within the Criminal Justice System. To date, there is a paucity of evidence assessing the effectiveness of interventions for offending behaviour in adults with ASD but without co-occurring intellectual disability (ID) across a broad range of forensic settings. The lack of robust evidence is concerning, as limited effectiveness may contribute to an increased likelihood of prolonged incarceration, particularly in the most restrictive settings. A PRISMA systematic review was conducted with a narrative synthesis to: (a) evaluate the evidence of the effectiveness of interventions aimed at reducing recidivism, (b) assess whether the core features of ASD impact the effectiveness of these interventions, and (c) identify additional factors that may affect the effectiveness of interventions within this population. Seven studies involving ten male participants were identified. The findings suggest that interventions for offending behaviours in adults with ASD without intellectual disability (ID) are largely inadequate, and that core ASD features need to be considered. Additionally, a complex interplay of risk factors potentially impacting intervention effectiveness was suggested. Limitations include heterogeneity across intervention types, measures of effectiveness, and what constitutes effectiveness. Despite the limited number of studies and data quality, the review aligns with a growing body of literature highlighting vulnerability and a need for evidence-based interventions for people with ASD. The review also discusses the broader implications of ineffective interventions.

**Supplementary Information:**

The online version contains supplementary material available at 10.1186/s40359-024-01770-1.

## Introduction

Autism spectrum disorders (ASD) represent a group of complex and highly heterogeneous neurodevelopmental disorders. A diagnosis of ASD is based on the presence of two core features: impairments in social communication and interaction (SCI), and restrictive and repetitive behaviours (RRBs) [1].

Phenotypic manifestations of the core features often present with varying degrees of social disengagement, difficulties in establishing and sustaining relationships, social naivety, lack of eye contact, and difficulties in interpreting facial expressions [2]. RRBs manifest as intense and highly restrictive special interests, a strong inclination for environmental consistency [3], cognitive rigidity, and hyper-or hypo sensory responses to the environment [4].

Additional factors modulate and influence these core features, including the extent of sensory and motor impairments, language and cognitive abilities, adaptive functioning, gender and the presence of co-occurring psychiatric disorders [5–7]. The increasing recognition of ASD has resulted in significantly higher diagnosis rates across all age groups [8], which are currently estimated to be 1 in 57 in England [9]. Consequently, this increase in diagnoses has led to a greater representation of individuals with ASD within the criminal justice system (CJS).

### ASD in the criminal justice system

An increasing body of research has highlighted the significant vulnerability experienced by individuals with ASD while navigating the CJS. This vulnerability becomes evident throughout multiple stages of the criminal justice process, ranging from initial encounters with police [10] through to police interviews [11], to court room proceedings [12] and prison services [13]. This heightened vulnerability is exacerbated by the reported general lack of understanding of ASD within the CJS, among both professionals and the general public [13–16].

Individuals *with* ASD and co-occurring intellectual disability (ID) are often identified and diverted from the criminal justice system (CJS). This is due to a recognition of their reduced culpability, a result of impairments in both intellectual and adaptive functioning [15]. In contrast, individuals with ASD but *without* co-occurring ID, the population on which this review focuses, exhibit significant deficits in adaptive functioning despite their intellectual capabilities. This difference is often referred to as the IQ functioning gap and is unique to individuals with ASD [17]. Despite impairments in adaptive functioning, this population is considered intellectually capable. Therefore, they are generally perceived as culpable and sufficiently competent to navigate the complexities of the CJS and receive a fair trial. This contrast raises further questions concerning culpability ranging from criminal responsibility to the appropriateness of sentencing.

Following conviction, when an offence has met the custody threshold, offenders with ASD are typically diverted to the community or prison. Alternatively, if detained under the Mental Health Act 1983 (the legislative framework governing mental healthcare and treatment in England and Wales), they may be detained in a secure hospital environment (classified as low, medium, or high security).

Estimating the prevalence of ASD within the UK prison population is difficult because of a lack of routine assessment; nonetheless, ASD is estimated to range between 1% and 4.4% [5]. Research has shown a disproportionately high prevalence of ASD in secure hospital settings (6.5%), exceeding the estimate for the general population [18].

Qualitative studies examining the experiences of prisoners with ASD without co-occurring ID have highlighted their increased vulnerability to bullying, exploitation, and social anxiety in prison [13]. In addition, research aimed at evaluating the prevalence of the broader autistic phenotype among a prison population, as well as comparing their mental health characteristics to those without, revealed a significant risk of self-harm and suicide in individuals presenting with autistic traits. Within this cohort, of the 240 prisoners assessed, 46 displayed significant autistic traits, with 12 meeting the diagnostic criteria for ASD. Notably, only two of these individuals had been previously recognised by the prison as having ASD. This finding highlights the under recognition of ASD and emphasises the heightened vulnerability of this population to a range of mental health risks within the prison environment [5].

Although it may be logical to assume that a secure hospital setting may better meet the treatment needs of people with ASD than a prison setting, current evidence suggests otherwise. Concerns have been raised, including the high likelihood of long-term seclusion in people with ASD compared to those without ASD [19] and significantly longer than average stays within secure hospital settings [20].

Despite several initiatives aimed at improving the recognition of ASD within the prison population [21], a recent UK government report on ‘neurodiversity’ [22], a term encompassing various conditions that fall into the broader category of neurodevelopmental disorders (NDDs) including ASD, highlighted three notable areas of concern. These included a greater likelihood of neurodivergent individuals being held on remand, inappropriately pleading guilty, and judges often failing to recognise a defendant’s neurodivergence as a mitigating factor when sentencing. These findings demonstrate that much work is needed to address the challenges faced by individuals with ASD and neurodivergent conditions in the CJS.

### ASD and risk of offending

While there is insufficient evidence to suggest that individuals with ASD are at greater risk of engaging in offending behaviours [23], it has been suggested that the core features of ASD may contribute to the risk of offending behaviours [24, 25]. Risk factors for offending behaviour in the general population are associated with the cumulative influence of various factors, including alcohol and drug abuse, low socioeconomic status, mental disorders, adversity, child abuse, and traumatic brain injury [26–28]. Less is known about the risk factors for offending behaviour within the ASD population, with the exception of co-occurring psychiatric disorders, such as personality disorders and psychosis [5].

Research suggests that individuals diagnosed with ASD early in life face barriers to services throughout their lifespan, resulting in unmet education, health, and therapeutic needs [29, 30]. Research suggests that certain demographic groups, such as women [31, 32], individuals from ethnic minorities, and those from lower socioeconomic backgrounds [9, 33], are far more likely to be underdiagnosed. This in turn increases the risk of unmet needs [34, 35]. These factors may contribute as variables that collectively increase the overall cumulative risk of engaging in offending behaviours.

### Forensic interventions

Interventions for offending behaviour often use cognitive-behavioural techniques to reduce recidivism, with an emphasis on perspective-taking, self-and relationship management, and problem solving. In the United Kingdom, the Ministry of Justice requires a sufficient evidence base for the accreditation of forensic interventions. This accreditation aims to promote high-quality programs in prisons and community settings to reduce recidivism [36].

Cognitive behavioural therapy (CBT) is widely recognised as one of the most effective interventions for offending behaviours [37]. There is evidence that CBT reduces recidivism by 20–30% in the general offending population [38, 39]. However, there is little evidence to support the effectiveness of such interventions for offending behaviour in forensic secure settings, often yielding inconsistent findings [40].

Beyond forensic settings, evidence suggests that adapted CBT is effective for individuals with ASD [41, 42]. These adjustments are necessary due to the core features of ASD and challenges in areas such as perspective taking and cognitive rigidity, both of which are conducive to successful therapeutic outcomes in this population [43]. Additionally, evidence supports the use of social skills training [44] and group-based social skills interventions in adults with ASD [41] However, there is no consensus regarding the specific adaptations most beneficial for individuals with ASD.

Furthermore, the lack of appropriate outcome measures has been reported to be a barrier to determining the effectiveness of interventions within secure forensic hospital settings [45–47]. Despite the evidence for CBT use within the general offender population and for individuals with ASD *outside* forensic settings, there are reports that the implementation of these interventions is not effective for individuals detained within secure hospital settings [19, 48, 49].

The increasing recognition of the vulnerability of individuals with ASD within the CJS highlights the urgent need for a systematic evaluation of the effectiveness of interventions for offending behaviours in adults with ASD. While previous research has examined interventions for individuals *with* ASD and co-occurring ID [49], a significant research gap remains regarding the effectiveness of forensic interventions for individuals with ASD but *without* co-occurring ID [14].

This systematic review aims to address this gap by conducting a comprehensive evaluation of intervention effectiveness in an ASD population *without* co-occurring ID.

### Research aims

This systematic review is guided by the following research objectives:

1. To systematically review and evaluate the effectiveness of interventions for offending behaviours in adults with ASD *without* co-occurring ID, as reported in the literature;
2. To ascertain whether the core features of ASD impact the effectiveness of the identified interventions; and.
3. To identify additional risk factors that may impact the effectiveness of interventions in this population.

## Method

### Inclusion criteria

Each potentially eligible study was screened based on the inclusion and exclusion criteria described in the PICO framework below [50].

- Population.

Participants included adults aged 18 years and older diagnosed with ASD, as defined by the authors in the literature. Studies involving participants with co-occurring ASD and ID and those that did not differentiate between these two populations were excluded.

- Intervention & Outcomes.

Our review aimed to identify studies that objectively and/or subjectively measured the effectiveness of therapeutic or pharmacological interventions for reducing recidivism in individuals with ASD exhibiting offending behaviours. These included interventions delivered in all categories, namely, prisons, probation supervision, and secure hospitals.

- Study Design and Comparison.

All primary research studies were included, regardless of publication date or country of origin. Studies that were peer-reviewed (e.g., grey literature and conference abstracts), systematic reviews, and those not published in English were excluded. An inclusion-exclusion criterion related to the type of comparison conducted in individual studies was not imposed.

### Search strategy

The search was conducted on the 27th of March 2021 across five databases, covering a broad timeframe and utilising international terminology. The databases included:

1. Embase (1974 to 2021).
2. Ovid MEDLINE(R) and Epub ahead of print, In-process, In data-review and other Non-Indexed Citations.
3. Ovid MEDLINE(R) Daily.
4. Global Health (1973 to March 2021).
5. APA PsychInfo (1806 to February 2021).

Furthermore, a web-based search using Google Scholar was conducted with the same search terms. The first 15 pages of results were manually reviewed; however, no additional studies meeting the inclusion criteria were found. Additionally, the reference lists and citations of relevant reviews were manually checked, but this did not yield any further eligible studies.

### Data selection and extraction

The data selection and extraction processes consisted of two stages:

During Stage 1, potential eligible studies were screened based on their titles and abstracts against the predefined inclusion and exclusion criteria. Owing to the limited number of results, the screening process was performed manually and repeated one week later to increase accuracy.

Stage 2 involved a comprehensive review of the full texts of the selected studies to confirm their alignment with the inclusion criteria. Relevant data were extracted and organised into spreadsheets using Microsoft Excel.

**Fig. 1:**
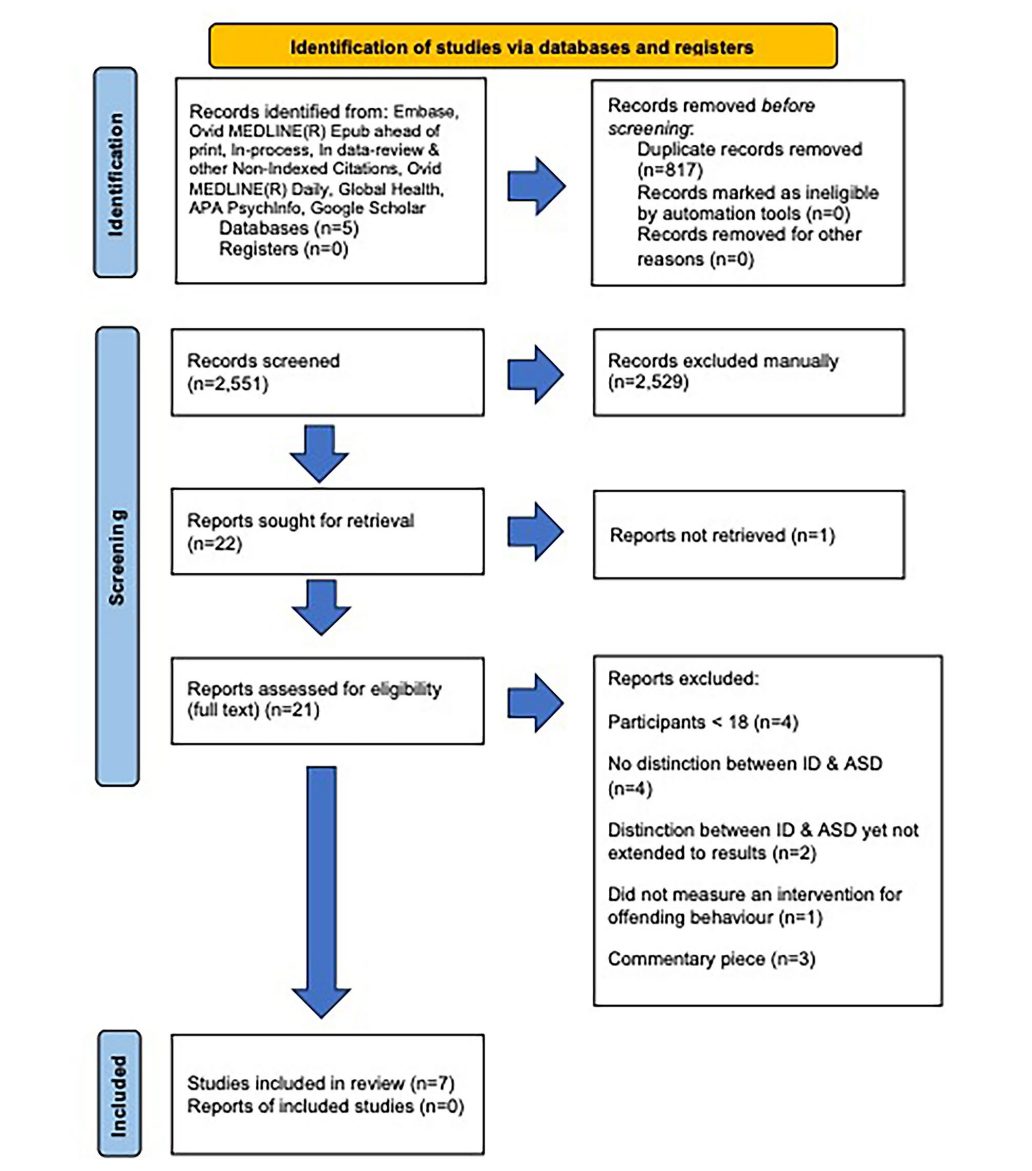
PRISMA flow diagram of searches of databases and registers only

### Data items

Consistent with the primary aim of this systematic review, the first outcome measure is the effectiveness of the identified forensic interventions, measured by a reduction in recidivism. While reducing recidivism is the principal goal of forensic interventions, it is often viewed as a proxy measure that may not fully capture the complexity of offending behaviours, particularly in cases of crossover crime [46, 51]. To address this limitation, additional relevant measures contributing to reduced recidivism were collected to allow for a preliminary assessment of intervention effectiveness. These additional measures included variables such as a reduction in security levels within institutional settings (i.e., medium to low security) or significant positive changes compared to baseline measurements recorded before and after the intervention.

The second aim of this review was to examine whether the core features of ASD present barriers to the rehabilitation process. To achieve this objective, data concerning the interactions between impairments in social communication and interaction (SCI) and restrictive and repetitive behaviours (RRBs) in relation to interventions within individual studies, as described by clinicians were collected and analysed.

Thirdly, this review aimed to identify additional risk factors described within findings that may influence the effectiveness of the interventions. The aim of the analysis is to provide a more comprehensive understanding of collective risk factors and their interactions with intervention effectiveness assessed through narrative synthesis. In addition, the data collected included the study design, author, and country of origin. When reported, participant demographics, such as age, gender, offence, ethnicity, and socioeconomic status, were reported. The intervention data included the type of intervention used, setting, duration, and frequency, only when available.

### Study risk of bias assessment

The Mixed Methods Appraisal Tool (MMAT) [52] is a comprehensive tool for critically evaluating various research methods. The methodological quality of each study and the potential risk of bias were assessed using the MMAT. The results of this assessment are presented in tabular form (Table 2, ‘MMAT Quality Appraisal’, appendix).

### Synthesis method

A narrative synthesis [53] was used for this review as a meta-analysis was not appropriate because of the significant heterogeneity between studies. The synthesis process began with a preliminary analysis, in which the data were extracted and presented in tabular form to provide a summary of the findings and to identify potential patterns within the data. A guided conceptual framework was constructed based on the narrative synthesis of the primary data. This framework aimed to assess both the similarities and differences between the included studies while exploring emerging thematic elements.

## Results

### Study selection

The initial database search returned 2,551 results after removing duplicates, as shown in Fig. 1 of the PRISMA flow diagram, which depicts the flow of information at each stage of the systematic review search. Subsequent screening included an initial assessment of the titles and a subsequent assessment of the abstracts, which led to the exclusion of an additional 2,530 articles. To ensure accuracy, abstract screening was repeated one week later. Subsequent full-text eligibility screening excluded 14 additional studies. The reasons for exclusion included the following: (a) participants under 18 years of age (*n* = 4), (b) lacked differentiation between the ID and ASD populations (*n* = 4), (c) were differentiated but not described in the context of the results (*n* = 2), (d) measurement of interventions for self-harm and suicide among offenders with ASD rather than for offending behaviour (*n* = 1), and (e) removal of commentary papers (*n* = 3). Consequently, the final number of included studies from the initial database search was seven (*n* = 7).

### Study characteristics

Among the seven studies identified, three were case reports (*n* = 3), two were qualitative studies (*n* = 2), and two were quantitative case series (*n* = 2). These studies jointly assessed the effectiveness of the various interventions. The total sample size of all the studies was limited to 10; all the participants were men, and demographic information was limited. It is worth noting that despite the use of international terminology in the search criteria, all seven articles described studies conducted exclusively in southern England, United Kingdom (UK). In these studies, all participants, apart from one were held in secure hospital units under the provisions of the Mental Health Act 1983. The most prevalent types of offending behaviours observed were sexual offences (*n* = 4), followed by manslaughter (*n* = 3), and arson (*n* = 3).

Table 1 ‘Summary of Findings’ provides a summary of each study included in the systematic review. The summary includes author information, available participant demographics, offence type, setting, detainment status (i.e., under the mental health act), intervention approach, study findings, intervention effectiveness, measurement used to assess effectiveness, and whether there was evidence to suggest that the core features of ASD impacted the effectiveness of forensic intervention(s). These are separated by impairments in social communication and interaction (SCI) and restrictive and repetitive behaviours (RRBs).

**Table 1:** Summary of findings

Author, Study Design	Participant, Demographic information	Offence	Setting, MHA Status	Intervention Approach	Study Findings	Intervention Effective	Measurement used to access Effectiveness	Evidence of ASD Impact Upon Intervention Effectiveness
Milton et al., 2002 [54] Case-report	*n* = 1 Male 30 s	Sexual offence	Secure hospital unit (Personality disorder) Under Mental Health Act (1983)	Cyproterone acetate (low compliance issue). Fluoxetine 60 mg daily. Psychological therapy -initially group sessions followed by individual sessions. Educational program, CBT, Art therapy, Reflective periods with staff.	Individual was moved to a different unit. Authors highlight that those with ASD tend to have negative outcomes in conventional forensic units.	**No**	MSI, BSI longitudinally, Fluoxetine 60 mg measured against self-report & inappropriate staring on secure unit.	**Yes** - Intensity of RRBs & SCI led to difficulties with group therapy, difficulties with staff interaction, forming connections with other patients. Considered vulnerable to exploitation by other patients.
Radley et al., 2011 [55] Case-report	*n* = 1 Male, 20 s	Arson	Low secure hospital unit Under Mental Health Act (1983)	Psycho-educational program. Occupational therapy for life skills, individual work addressing substance misuse/fire setting. Medication for alcohol-induced psychosis.	Offending a result of ASD symptom – RRBs (fire), SCI - led to social isolation, in turn alcohol abuse. Late diagnosis potentially increased risk of offending.	**Yes** Understood offending & prevention of offending. Improved SCI strategies. Accepted ASD diagnosis, medication & continued support. following two years of interventions	Move through levels of security – to a less secure setting.	**Yes** - SCI led to initial difficulties with group work.
Murphy 2010., [56] Case-report	*n* = 1 Male 20 s	Manslaughter	High secure hospital unit Under Mental Health Act (1983)	Individual adapted CBT –skills development – emotional recognition, problem solving, understanding of consequences, victim empathy, interpersonal conflict & anger expression. Education & occupational therapy. Medication for psychosis.	Features of ASD played role in offending, late diagnosis increases risk. Standard approach & risk assessment unsuitable for individuals with ASD.	**No** After 4 years of admission & over 70 h of individual contact. Psychosis alleviated with medication.	STAXI II – outwards anger reduction yet inwardly high. Lack of empathy, no acceptance of responsibility -determined by interview.	**Yes** – SCI – lack of interaction with staff & other patients, social isolation, difficulties with empathy. RRBs - cognitive rigidity.
Langdon et al., 2013 [57] Quantitative case series	*n* = 4 Males	Manslaughter *n* = 2 Arson *n* = 2	Secure hospital unit for individuals with ID Under Mental Health Act (1983)	Adapted version of Equipping Youth to Help One Another (EQUIP) for intellectual or developmental difficulties – non offence specific- 12-week program, 4 x per week	Researchers suggest EQIP could be a promising intervention for ID and developmental disabilities	**No** Not for intended purpose, author report *n* = 3 benefitted socially with intervention, yet advised caution interpreting findings.	Data collected beginning & end of treatment. Sociomoral reflection (SRM-SF), problem solving ability (PST), cognitive distortions (HIT) & anger	n/a
Murphy et al., 2007 [58] Quantitative case series	*n* = 2 Males	Sexual offences	*n* = 1 - ID Secure hospital unit Under Mental Health Act (1983) *n* = 1 in the community – lived alone	Simplified form of CBT using themes of A-SOTP developed for people with ID who sexually offend.	ASD may be a risk factor to recidivism in men with co-occurring ID	**No** ASD participant repeated one year program twice. Both reoffended. Further details not clear due to lack of distinction.	Improvement to baseline data at group level on Sexual Knowledge (SAKS). Victim empathy (VES-A). Questionnaire on attitudes w sexual offending QACSO although not in sexual offender’s self-appraisal scale (SOSAS) - cognitive distortions	**Yes **– SCI difficulties with theory of mind and victim empathy
Melvin et al., 2020 [59] Qualitative study	*n* = 10 Group facilitators’ views	Sexual offences	Secure hospital unit & in the community	A-SOTP – adapted sex offender treatment program.	Overall clinicians viewed many aspects of the A-SOTP inappropriate across various domains for those with ASD	**No** For intended purpose but increased opportunities to improve social skills in a group setting	Clinicians’ judgement of patient	**Yes** – SCI emotion recognition & regulation, cognitive inflexibility, lack of empathy, lack of desire to adhere to social rules
Melvin et al., 2019 [60] Qualitative study	*n* = 1 Male	Sexual offences	Secure hospital unit Under Mental Health Act (1983)	A-SOTP – adapted sex offender treatment program)	Participant views of A-SOTP treatment & risk reduction - unclear for ASD without ID participant. Author recognises features of ASD complicates effectiveness of this Intervention.	**No** ASD only participant completed program on 6 occasions, yet reoffended	Recidivism & participant views on treatment & risk reduction	**Yes** – SCI social exclusion, perceived lack of internal control

### Risk of bias in studies

The methodological quality of the studies was assessed using the MMAT [52] (Table 2, ‘MMAT Quality Appraisal’, appendix). Each of the three case reports received a 3-star rating, indicating a moderate risk of bias and meeting 75% of the qualitative MMAT criteria [54–56].

The two quantitative case series were found to be at a higher risk of bias due to difficulties in distinguishing the treatment groups, recruitment difficulties, lack of a control group, and incomplete outcome data for the ASD group without co-occurring ID. They received a 2-star rating, meeting 50% of the MMAT quantitative criteria [58].

The second quantitative study [57], raised concerns about the validity and reliability of outcome measures, which were originally designed for the ID population but applied to the ASD group without co-occurring ID. This study also received a 2-star rating and met 50% of the MMAT’s quantitative criteria.

The remaining qualitative studies received a 3-star rating, meeting 75% of the MMAT criteria. The first evaluated intervention effectiveness from the perspective of the clinicians who delivered the therapeutic program [59]. The second assessed offenders’ views via self-report, which carry a potential risk of response bias [60].

Selection bias was observed in studies that combined ID and ASD populations. Overall, it was difficult to establish a causal relationship between the interventions and outcomes.

Notably, not all the studies reviewed explicitly documented obtaining informed consent from participants. The discrepancy in informed consent between studies, particularly in restrictive forensic settings, presents challenges extending beyond ethical considerations. Such discrepancies may compromise the validity of intervention comparisons, introduce biases in participant selection, and undermine the reliability of data.

### Interventions

The interventions examined across the reviewed studies were diverse, as presented in Table 3, titled ‘Summary of Interventions’.

**Table 3:** Summary of interventions

Intervention	Offence Type	Mechanism of Action/ Theory	Studies using this intervention	Evidence Base for use	Measurements used to assess effectiveness
**Pharmacological**					
Cyproterone Acetate	Sexual Offending	Testosterone inhibitor.	Milton et al., 2002 [54]	Meta-analysis has since suggested that the limited evidence supporting the use of Cyproterone Acetate in sexual offenders is not sufficient to guide practice [61].	Self-report, adherence concern.
Fluoxetine 60 mg	Sexual offending (off-label)	SSRI – blocks reuptake of serotonin.	Milton et al., 2002 [54]	Evidence based on 3 observational studies; clinical significance not fully determined [62].	Self-report and clinician assessed no reduction of inappropriate glancing behaviours. Significant negative side effects.
Antipsychotic (Unspecified)	Arson (alcohol induced psychosis)	Modifies dopaminergic neurotransmission.	Radley et al., 2011 [55]	A systematic review found evidence for antipsychotics for alcohol induced psychosis inconclusive [63]. ASD affects outcomes in psychosis treatment [64].	Clinician assessment, decrease in delusions.
**Psychological**					
A-SOTP (adapted sex offender treatment program)	Sexual offending	Aim is to increase victim empathy in sex offenders, address cognitive distortions and the reduction of offending attitudes [60].	Murphy et al., 2007 [58] Melvin et al., 2020 [59] Melvin et al., 2019 [60]	Despite lack of RCTs, evidence suggests A-SOTP is beneficial for individuals with ID [65]. No evidence of benefit within ASD population.	Murphy et al., 2007 [58] – sexual attitudes SAKS, attitudes consistent with sexual offending QACSO, sexual offenders’ self-appraisal (SOSAS), victim empathy (VES-A). Melvin et al., 2019 [60] - offender self-report. Melvin et al., 2020 [59] – Clinician views.
EQUIP (adapted version of Equipping Youth to Help One Another for intellectual or developmental difficulties)	Sexual offending	Based on theory that moral reasoning is underdeveloped in young offenders and individuals with ID and DD (Langdon et al., 2013) [57].	Langdon et al., 2013 [57]	EQIP has been beneficial to young offenders therefore may be beneficial for ID & ASD (Landon et al., 2013) [57].	Pre & post scores on moral reasoning, cognitive distortions, problem solving abilities and anger.
CBT (adapted cognitive behavioural therapy)	All offending behaviours	Identifies maladaptive thoughts and beliefs with the aim of altering thought patterns. Studies aimed to address the following: Skills development, consequences of actions, victim empathy, acceptance of ASD diagnosis, addressing executive functioning difficulties, and interpersonal conflict.	Milton et al., 2002 [54] Murphy., 2010 [56] Radley et al., 2011 [55]	Evidence suggests CBT can be helpful for those with ASD, although further research is needed to determine most effective adaptations which are not standardised [42] This evidence does not extend to forensic settings for individuals with ASD.	Milton et al., 2002 [54] adapted items from Behavioural Status Index. Radley et al., 2011 [55] unspecified. Murphy., 2010 [56] self-report, clinician view.
**Supplementary Interventions (Non forensic specific)**					
Occupational Therapy	All offending behaviours	To improve life skills, independence.	Murphy., 2010 [56] Radley et al., 2011 [55]	N/A	Could not be ascertained.
Speech & Language Therapy	All offending behaviours	To improve communication skills.	Radley et al., 2011 [55]	N/A	Improved communication within unit.
Art Therapy	All offending behaviours	To improve self-exploration and expression.	Milton et al., 2002 [54]	N/A	Could not be ascertained.

Three studies incorporated both pharmacological and psychological interventions. Specifically, antipsychotics were used to address co-occurring psychosis, contributing to instances of offending behaviour [55]. Antipsychotics were also used to manage stress-induced psychosis [56]. In the context of directly treating offending behaviours, two distinct medications were applied in cases of sexual offending, each with different mechanisms of action [54] (Table 3).

Four studies relied exclusively on psychological interventions [57–60]. Among these, two studies implemented adapted forms of CBT. Specific details regarding the non-standardised adaptations used in CBT were not provided by the study author, except that individual delivery was necessary due to difficulties encountered within group settings [54, 56].

The third study that incorporated CBT included elements similar to those of the Adapted Sex Offender Treatment Program (A-SOTP) [58]. The effectiveness of the A-SOTP was described in two studies [59, 60]. Furthermore, the Equipping Youth to Help One Another (EQUIP) was adapted and piloted for use with individuals with ID and developmental disabilities (DD) who had committed sexual offences [57]. Supplementary interventions included speech and language therapy to facilitate communication [55], occupational therapy to address impairments in executive functioning [55, 56] and art therapy [54].

Table 3 visually depicts a summary of the diverse interventions extracted, reviewed, and categorised according to intervention type: pharmacological, psychological, and supplementary intervention approaches. In addition, the table includes the type of offence, studies using intervention, underlying mechanism of action or theory, evidence base supporting intervention, and measurements used to assess effectiveness.

### Measurements

Numerous approaches were adopted to measure effectiveness across the studies. Two studies measured effectiveness by reduced recidivism and the need to repeat the intervention. Other studies utilised a range of standardised measurements to evaluate psychological interventions. For example, one study [54] employed the Behavioural Status Index (BSI) every six months as a measurement tool. In contrast, another [56] employed the State Trait Anger Expression Inventory (STAXII II) and the Millon Multiaxial Personality Inventory (MMPI), combined with standardised risk assessment, one-year postintervention.

Regarding pharmacological interventions, one case report used a combination of subjective and objective measurements. These included self-reports and the systematic monitoring of inappropriate glancing behaviours over time by staff members [54]. In another instance, the reduction in verbalised delusions served as a measure of the effectiveness of antipsychotic medication [55, 56].

The effectiveness of interventions such as the A-SOTP was assessed differently across the two studies. In one study, effectiveness was evaluated through clinician views [59], while in the other, effectiveness was determined by the participants’ subjective experiences with the intervention [60].

In the case of CBT, which shares similarities with A-SOTP, standardised measures were applied both pre- and post-intervention. These measures consisted of sexual attitudes consistent with sexual offending (QACSO), sexual offenders’ self-appraisal scale (SOSAS), the sexual attitudes and knowledge scale (SAKS), and the victim empathy scale-adapted (VES-A) [58].

The EQIP study, which also focused on sexual offending [57], assessed effectiveness by examining improvements in baseline scores on standardised tests related to moral reasoning, cognitive distortions, problem-solving abilities, and anger. In addition, a move to a lower security level was considered an indicator of overall effectiveness. Furthermore, in a case study that included speech and language therapy, the clinician’s subjective view of improved communication within the secure unit served as a measure of the intervention’s effectiveness [55].

### Outcomes

Among the seven studies reviewed, only one pertaining to an arson offence considered the intervention(s) effective. In this case, a pharmacological intervention was used to treat co-occurring alcohol-induced psychosis, and the unspecified antipsychotic proved successful in reducing delusions. Furthermore, speech and language therapy aimed at improving communication skills was also deemed to be effective [55].

However, the remaining six studies, which included a total of nine participants, concluded that the interventions were largely ineffective. One case report addressing sexual offending behaviours used pharmacological interventions. The first involved cyproterone acetate, a testosterone inhibitor, however, the outcome could not be conclusively determined owing to adherence and dosage issues [54]. In the second, the selective serotonin reuptake inhibitor (SSRI) fluoxetine was deemed ineffective, as inappropriate behaviours did not significantly decrease [54]. It is worth noting that the evidence for both of these drugs has since been described as insufficient to guide clinical practice, with cyproterone acetate considered inadequate [61], and the evidence for fluoxetine has not been fully determined [62].

Among the two studies that utilised the A-SOTP and a similar form of CBT for sexual offending, one participant repeated the intervention program six times and subsequently re-offended and a further two participants repeated the yearlong intervention program and reoffended [58]. These findings are consistent with the results of the study that assessed clinician views [59]. Even in the case of CBT, as used in two studies, the intervention was deemed ineffective despite adaptations made to accommodate individuals with ASD [54, 56].

### ASD core features and impact upon intervention effectiveness

**Fig. 2:**
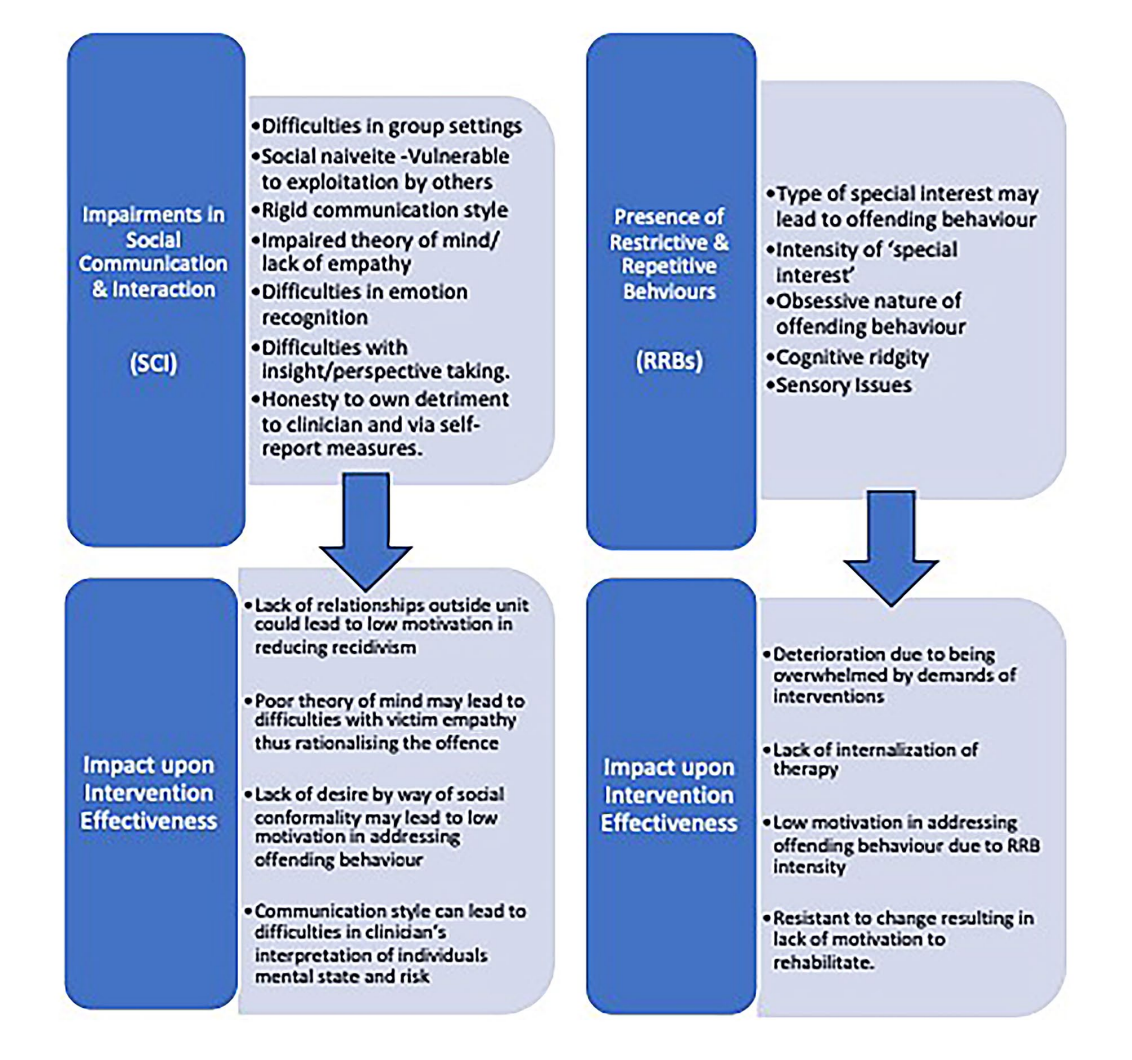
Impact of The Core Features of ASD upon Intervention Effectiveness. *Note. This describes the core features of ASD, both ‘impairments to SCI’ and ‘presence of RRBs’, and their impact upon intervention effectiveness as extracted from studies*

The application of a narrative synthesis facilitated the identification and extraction of recurring patterns within the data. These patterns were evident across all the studies, highlighting the considerable challenges posed by impairments in social communication and interaction (SCI) and the presence of restrictive and repetitive behaviours (RRBs) on the effectiveness of interventions, as depicted in Fig. 2.

### Additional factors impacting intervention effectiveness

In addition to the core features of ASD, this review sought to identify additional risk factors that may influence the effectiveness of the intervention(s). Potential risk factors highlighted by the authors of each study were collected, and through narrative synthesis, several recurring themes emerged from the data. Co-occurring personality disorders and psychosis [55, 56], were identified as potential factors impacting intervention effectiveness, as described within the literature. Additionally, events such as childhood adversity, sexual abuse, trauma, and having a dysfunctional family life were described as potential contributors [58]. Late diagnosis of ASD was theorised to lead to maladaptive coping skills deriving from unmet needs, which were described in three of the studies [54–56].

An overarching theme identified across the majority of the seven studies was the insufficiency of service provision, staff expertise, and the evidence base.

## Discussion

The present systematic review identified seven studies with ten participants who underwent forensic interventions aimed at reducing offending behaviours in adults with ASD, particularly those *without* co-occurring ID. The principal aim of this review was to evaluate the effectiveness of these interventions. The secondary aim was to examine whether the core features of ASD have an impact on the effectiveness of these forensic interventions and to identify other variables that may impact the overall effectiveness of interventions.

Regarding the first aim, the evidence suggests that the interventions reviewed were inadequate. However, these findings should be treated with caution not only because of the small sample size but also because of limitations in the generalisability of the findings. Despite an extensive literature search, all the studies were conducted in southern England, UK, and included only male participants. In addition, all participants, with the exception of one individual living in the community, were detained within secure hospital settings under the provisions of the Mental Health Act (1983). This highlights the lack of data from prison and the probation service, which limits the scope of the review. Furthermore, this review highlights a critical lack of research within this domain. Even when the literature was identified, it was often of inadequate quality owing to various design limitations. The significant heterogeneity between studies, each utilising distinct intervention methods and tools for measuring intervention effectiveness, illustrates a notable lack of standardisation in both clinical and research methodologies within this field. This lack of consistency aligns with broader research on mental health in individuals with ASD [45, 46]. Nonetheless, the forensic domain faces additional challenges, such as the lack of randomised control trials, which means that the effectiveness of interventions is difficult to fully determine. These challenges are exacerbated by unavoidable confounding variables, the risk of bias, and the ethical implications of a no-treatment group [66], all of which contribute to the lack of evidence.

The secondary aim was to examine the potential impact of the core features of ASD on the effectiveness of interventions designed to reduce recidivism. The data patterns identified through narrative synthesis consistently emerged across all studies, highlighting the significant challenges posed by impairments in social communication and interaction (SCI) and the presence of restrictive and repetitive behaviours (RRBs). These challenges highlight the general inappropriateness of forensic interventions within this population.

The third and final aim was to identify factors, beyond the core features of ASD, that may influence the effectiveness of interventions. Throughout the studies, a recurring theme emerged, highlighting significant systemic factors impacting intervention effectiveness. These include issues such as a shortage of government funding leading to inadequate service provision, the question of whether ASD and ID services should be combined, and the substantial unmet needs throughout the lifespan of individuals with ASD, all of which affect the success of forensic interventions. While the core features of ASD are significant, they may not be the primary cause of intervention failure. Rather, they seem to be contributing factors within a broader and more complex array of variables that collectively impact the overall effectiveness of these forensic interventions.

### Implications

The inadequate provision of forensic services carries significant implications, especially when prolonged detainment becomes necessary due to the shortcomings of forensic interventions. Such deficiencies may subject individuals with ASD to non-evidence-based interventions, often repeatedly [56, 58]. This then increases the likelihood of these individuals being labelled as ‘unrehabilitated,’ potentially leading to extended periods of detainment. Consequently, this creates a counterproductive cycle that not only exacerbates the economic burden but also raises serious concerns about human rights and the potential legal consequences of prolonged confinement.

These issues underscore fundamental questions about the fairness and adequacy of the legal system. Therefore, addressing these knowledge gaps and the lack of evidence-based approaches are crucial to ensuring a more equitable criminal justice system for individuals with ASD.

### Future research

This review identifies several key areas for future research in this field. Developing evidence-based interventions tailored to the unique needs of individuals with ASD is crucial. Establishing a consensus on the measurements used for assessing the effectiveness of these interventions, as well as a clear definition of what constitutes effectiveness, would significantly enhance research quality.

Moreover, due to the bias towards studies conducted in southern England, the consistency of interventions for treating offending behaviours in adults with ASD in England remains unclear, especially considering the persistent regional health disparities between the North and South of England [67, 68].

### Electronic supplementary material

Below is the link to the electronic supplementary material.

Supplementary Material 1

## Data Availability

No datasets were generated or analysed during the current study.
