# Supplementary material for: The effectiveness of interventions for offending behaviours in adults with autism spectrum disorders (ASD): a systematic PRISMA review
Source: BMC Psychol. 2024 May 30;12:316. doi: 10.1186/s40359-024-01770-1 (PMC11140896; doi:10.1186/s40359-024-01770-1)
Supplement: Supplementary file 1 — Supplementary Material 1 [file 40359_2024_1770_MOESM1_ESM.docx]

**Appendices**

1. **Search Strings**

The initial search terms were selected to comprehensively identify literature pertaining to ASD. This strategy included both current and historical diagnostic labels that are longer in use. The second set of search terms were selected to include any type of offending behaviours and to identify literature across various diverse settings post-conviction among both adult and young adult populations in different settings. The third set of search terms were selected to capture a broad range of therapeutic and rehabilitative strategies aimed at reducing recidivism. This selection aimed to ensures a comprehensive search for both direct interventions and broader rehabilitative measures.

1. autis* OR ASD OR asperge* OR developmental dis* OR high functioning OR pervasive developmental* OR PDD.

AND

1. offend* OR prison OR crim* OR probation OR secure OR forensic OR jail OR juvenile detention OR youth detention OR custod* OR correction* OR incarcerat*.

AND

1. Treat* OR interven* OR therap* OR recidiv* OR rehabil*
2. **MMAT Table**

Table 2: MMAT Quality Appraisal (Hong et al., 2018)

| Author | Qualitative | Quantitative | Overall quality | Comments |
| --- | --- | --- | --- | --- |
|  | **1.1 1.2 1.3 1.4 1.5** | **4.1 4.2 4.3 4.4 4.5** |  |  |
| Milton et al., 2002 | Y Y Y Y Y |  | *** |  |
| Radley et al., 2011 | Y Y Y Y Y |  | *** | Unclear which intervention led to outcome as delivered simultaneously. |
| Murphy 2010 | Y Y Y Y Y |  | *** |  |
| Melvin et al., 2019 | Y Y N N Y |  | *** | Risk of response bias |
| Melvin et al., 2020 | Y Y Y Y Y |  | *** |  |
| Langdon et al., 2013 |  | Y Y N Y N | ** | Validity & reliability of measurements - used on ASD group developed for ID population. unclear if intervention led to outcome. |
| Murphy et al., 2007 |  | Y Y N Y Y | ** | Differences between treatment groups unclear. No control group, unclear if intervention led to outcome. Difficult to ascertain further details regarding ASD only participants. |

| * Meets 25% of MMAT criteria  ** Meets 50% of MMAT criteria | *** Meets 75% of MMAT criteria  **** Meets 100% of MMAT criteria |
| --- | --- |
